# Epigenetics, bacterial metabolites and type 2 diabetes: a new view of gut-genome interactions

**DOI:** 10.3389/fmicb.2026.1886562

**Published:** 2026-08-06

**Authors:** Aqeel F. Taqi, Ahmad Albuloushi, Ayat Saleh, Fouz Alqaderi, Taiba AlGharib, Lulwa Al-Abdullah, Fawaz Alzaid, Anwar Kandari

**Affiliations:** 1 Dasman Diabetes Institute, Kuwait City, Kuwait; 2 Ministry of Health, Kuwait City, Kuwait; 3 University of Essex, Colchester, Essex, United Kingdom; 4 Institut Necker Enfants Malades, Université Paris Cité, INSERM UMR-S1151, CNRS UMR-S8253, Paris, France

**Keywords:** bacterial metabolites, DNA methylation, epigenetics, SCFAs, type 2 diabetes

## Abstract

Type 2 diabetes (T2D) is traditionally viewed as a disorder of glucose homeostasis, yet growing evidence suggests that its pathogenesis is deeply influenced by interactions between the gut microbiota and host gene regulation. Bacterial metabolites have emerged as key molecular mediators capable of linking environmental exposures, metabolism and epigenetic control of gene expression. These insights are reshaping current understanding of how microbial signals contribute to metabolic disease. This review examines the emerging role of bacterial metabolites as modulators of host epigenetic landscapes in T2D. We discuss how short-chain fatty acids (SCFAs), particularly butyrate and propionate, regulate chromatin accessibility through histone deacetylase inhibition and changes in histone acetylation. Beyond SCFAs, we highlight additional classes of microbiota-derived metabolites, including tryptophan-derived indoles such as indole-3-propionic acid, polyamines such as spermidine and aromatic amino acid derivatives, that expand the microbiota–epigenetic axis through mechanisms involving DNA methylation, transcriptional regulation, mitochondrial function and inflammatory signaling. These effects occur in a tissue-specific manner across metabolically relevant organs including the colon, liver, pancreatic *β*-cells and adipose tissue, reflecting differences in metabolite exposure and cellular metabolic context. We further discuss emerging evidence suggesting that microbial metabolites may contribute to intergenerational metabolic programming, linking maternal diet, microbiota composition and offspring epigenetic trajectories. Finally, we examine therapeutic perspectives targeting this axis, including dietary strategies, microbiota-directed interventions and metabolite-based therapies. Collectively, discussed works support a conceptual shift in our understanding of T2D biology: gut microbial metabolites are active regulators of chromatin architecture and metabolic gene networks that influence the risk, development and progression of T2D. Understanding these gut-epigenome interactions may provide new opportunities to identify biomarkers of metabolic risk and to develop mechanism-based strategies for diabetes prevention and treatment.

## 1 Introduction

Type 2 diabetes (T2D), the most common form of diabetes, is one of the most significant health challenges of the 21st century. Increasing evidence indicates that interactions between the gut microbiota, microbial metabolites and host epigenetic regulation contribute to metabolic dysfunction and disease progression. These findings have highlighted the microbiota–epigenetic axis as an emerging area of investigation. Worldwide prevalence of T2D continues to rise (Mendis et al., 2025), driven by aging populations, sedentary lifestyles and dietary transitions (Bellary et al., 2021). The IDF Diabetes Atlas estimates that 589 million adults were living with diabetes in 2024, representing 11.1% of the global adult population, and projects this to rise to 853 million by 2050 (Duncan et al., 2025). More than 4 in 10 people remain undiagnosed, and the majority live in low- and middle-income countries. Prediabetes cases, including adults with impaired glucose tolerance (IGT) and impaired fasting glucose (IFG), are associated with a high risk of progression to T2D (Diabetes World Health Organization (WHO), 2024). In 2024, it was estimated globally that 634.8 million adults have IGT and 487.7 million adults have IFG. By 2050, both are projected to increase to 846.5 million and 647.5 million adults, respectively. The age-standardised prevalence in middle- and low-income countries was 12.3% and 9.5% in middle-income countries for IGT and IFG, respectively, and 11.6% and 6.7% in low-income countries for IGT and IFG, respectively (Duncan et al., 2025). The condition is associated with substantial morbidity and mortality due to micro- and macrovascular complications (Zakir et al., 2023), and comorbidities such as metabolic dysfunction–associated steatotic liver disease (MASLD) (Han et al., 2025). To address the growing burden, the mechanisms underlying risk and disease progression must be better understood. Although diabetes encompasses distinct disorders, this review focuses primarily on T2D, obesity and related metabolic dysfunction, with particular emphasis on microbiota-derived metabolites and their influence on host epigenetic regulation. Type 1 diabetes and other forms of diabetes involve distinct pathophysiological mechanisms and microbiota-associated signatures and are therefore beyond the scope of the current review.

Diabetes is associated with alterations in the gut microbiota and shifts in microbial metabolite profiles (Yu et al., 2025). The gut microbiome contributes to metabolic homeostasis through host–microbe interactions that influence glucose metabolism, insulin sensitivity and inflammation (Das et al., 2021; Que et al., 2021; Zhang et al., 2024). The precise mechanisms through which microbial signals translate into metabolic improvement or dysfunction remain incompletely defined.

Macroeconomic analysis estimates the 2020 to 2050 global economic cost of diabetes to be INT$10.2T, rising to INT$78.8T (INT$ referring to 2017 international $) including informal caregiving (Chen et al., 2026). Most burden is driven by complications and comorbidities, namely kidney failure treated by transplant or dialysis and lower-extremity amputations (Wang et al., 2022). Against this backdrop, the microbiome therapeutics sector is expanding, with market estimates rising from ~US$250M in 2025 to more than US$3.4B by 2034, reflecting growing translational investment in microbiota-directed strategies (Precedence Research, 2025; Mordor Intelligence, 2025). If microbiota-directed or related epigenetic therapies can safely improve insulin sensitivity and metabolic function, even modest effects could translate into substantial reductions in economic and long-term complication burden (Ur Rehman et al., 2025). Despite the rise in the prevalence of diabetes, its related complications, and the estimated costs, diabetes treatment has either not increased or has not increased sufficiently to keep pace with this rise, especially in low- and middle-income countries (Collaboration NCDRF, 1990).

Importantly, the gut microbiome is shaped by socioeconomic contexts, a known determinant of T2D susceptibility. Population studies indicate that socioeconomic position is associated with microbiome diversity and composition, and lower socioeconomic status is linked to distinct taxonomic profiles (Bowyer et al., 2019). Food insecurity is associated with alterations in gut microbial composition (Christian et al., 2020), and some effects are transmitted across generations. Such effects can be mediated epigenetically and can modify metabolic risk and response to intervention (Hill-Briggs et al., 2020).

Epigenetic regulation of gene expression has emerged as an interface linking environmental signals to metabolic regulation (Stilling et al., 2014). Epigenetics refers to the heritable reversible modifications, including DNA methylation, histone modifications and chromatin remodeling, that regulate gene transcription without altering the DNA sequence (Cedar and Bergman, 2009). These mechanisms are essential for maintaining pancreatic *β*-cell identity (Volkmar et al., 2019) and contribute to the regulation of metabolic pathways in tissues such as the liver and adipose tissue, where they influence inflammation, glucose metabolism and metabolic memory (Ling and Ronn, 2019).

Epigenetic pathways are highly responsive to cellular metabolic states. Many chromatin-modifying enzymes require metabolic intermediates as substrates or cofactors, creating a direct link between metabolic signals and gene regulation (Miro-Blanch and Yanes, 2019). Importantly, these epigenetic pathways function as integrative hubs that respond to multiple inputs, including nutrient availability, hormonal signaling, pharmacological agents, oxidative stress and cellular metabolic states. Microbiota-derived metabolites therefore represent one component of a broader regulatory network rather than the sole determinants of chromatin remodeling. Within this context, gut microbiota-derived metabolites have emerged as important contributors to host epigenetic regulation.

Bacterial metabolites, including short-chain fatty acids (SCFAs) (Hosseinkhani et al., 2021), tryptophan-derived indoles and polyamines (Zeng et al., 2025) and aromatic amino acid (AAA) derivatives (Siracusa et al., 2026) can directly and indirectly influence chromatin regulation. By modulating processes such as histone acetylation and DNA methylation, microbial products can reshape host chromatin states and transcriptional programs, thereby linking microbiota to metabolic regulation (Fernandes and Vinolo, 2024).

This review discusses how bacterial metabolites influence epigenetic regulation in T2D, including tissue- and cell-specific mechanisms, potential intergenerational implications and emerging microbiota-informed therapeutic strategies. Although numerous microbial metabolites have been implicated in host metabolism, this review focuses on SCFAs, tryptophan-derived indoles, polyamines and AAA-derived metabolites because they currently have the most substantial mechanistic and translational evidence linking microbial metabolism to epigenetic regulation in T2D. First, we examine both classical and emerging bacterial metabolites and their epigenetic actions, followed by their tissue-specific effects across metabolically relevant organs. We then discuss evidence for intergenerational metabolic programming and evaluate therapeutic perspectives targeting the microbiota–epigenetic axis. Finally, we highlight current conceptual and translational challenges, including the need to establish causal relationships and to determine the stability and reversibility of metabolite-driven epigenetic changes.

Literature discussed in this narrative review was identified through searches of scientific literature databases, such as PubMed and Scopus, supplemented by citation screening of relevant reviews and primary articles. Search terms included combinations of “type 2 diabetes,” “gut microbiota,” “microbial metabolites,” “short-chain fatty acids,” “indole-3-propionic acid,” “polyamines,” “epigenetics,” “DNA methylation,” “histone modification,” and “chromatin.” Emphasis was placed on English-language articles published primarily within the past 10–15 years, while older seminal studies were included where directly relevant to foundational mechanisms. Priority was given to studies in humans, human tissues, and experimental models of T2D or obesity-related metabolic dysfunction. Articles focused exclusively on type 1 diabetes, non-mammalian systems, or unrelated disease contexts were generally excluded unless they provided important insight relevant to microbiota-epigenetic interactions in T2D.

## 2 Beyond classical pathways: bacterial metabolites in type 2 diabetes epigenetics

### 2.1 Classical pathways: short-chain fatty acids (SCFAs)

**Figure 1:**
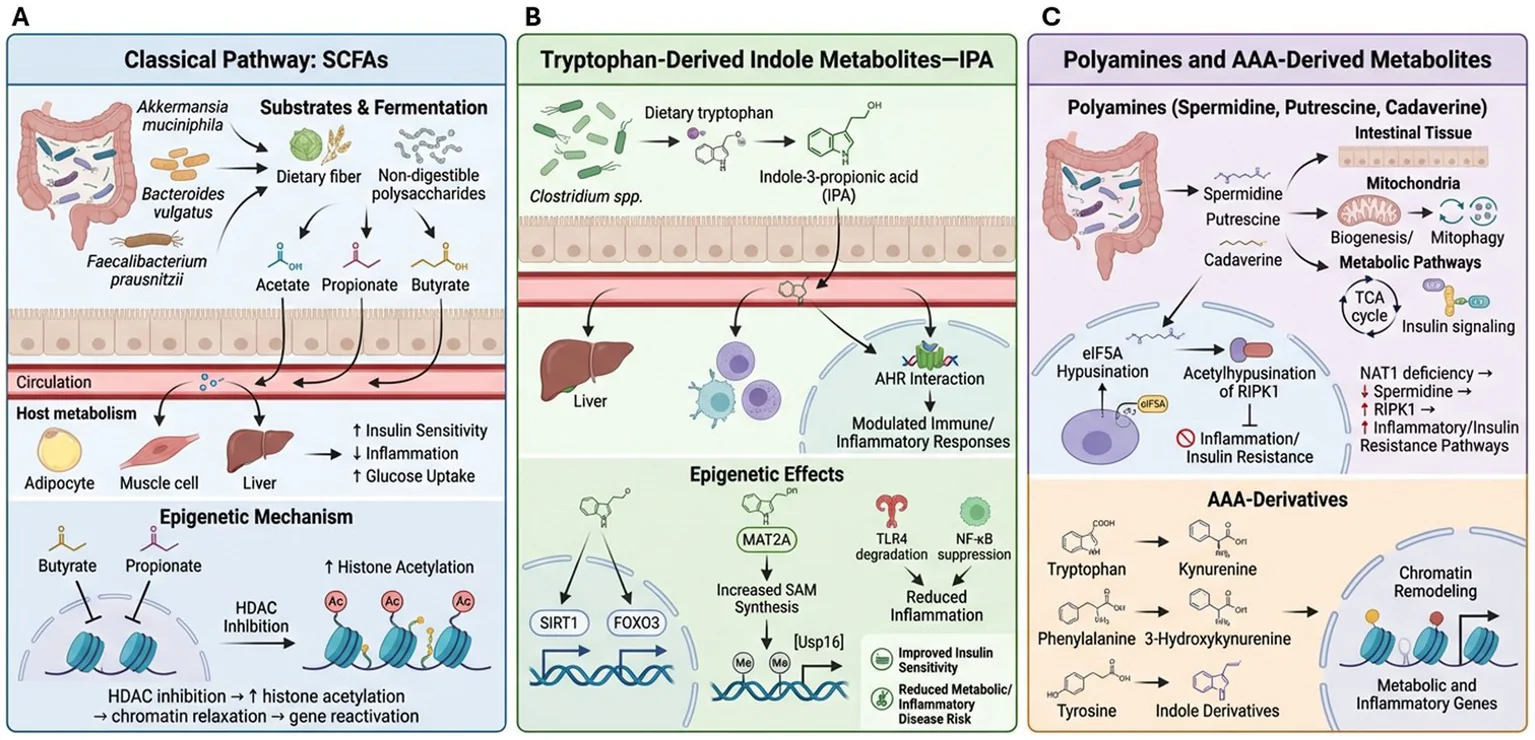
Gut microbial metabolites influence host metabolism and gene regulation through several pathways relevant to type 2 diabetes. **(A)** Fermentation of dietary fibers and non-digestible polysaccharides by gut bacteria such as *Akkermansia muciniphila*, *Bacteroides vulgatus* and *Faecalibacterium prausnitzii* generates the short-chain fatty acids (SCFAs) acetate, propionate and butyrate. These metabolites can enter circulation and inhibit histone deacetylases (HDACs) across different tissues. HDAC inhibition increases or maintains histone acetylation and promotes transcriptional programs associated with improved insulin sensitivity, increased glucose uptake and reduced inflammation. **(B)** Microbial metabolism of dietary tryptophan produces indole derivatives, including indole-3-propionic acid (IPA), which interact with host effector proteins, namely the aryl hydrocarbon receptor (AHR) that transcriptionally regulates multiple processes. IPA enhances acetylation at target loci, increasing expression of target genes sirtuin-1 (SIRT1) and forkhead box O3 (FOXO3) which control mitochondrial function and cellular stress resistance. IPA also activates methionine adenosyltransferase 2A (MAT2A) which increases S-adenylmethionine (SAM) synthesis, promoting DNA methylation at the gene coding the ubiquitin specific peptidase 16 (USP16). This degrades toll-like receptor 4 (TLR4) and suppresses nuclear factor kappa-light-chain-enhancer of activated B cells (NF-κB) signaling, leading to reduced inflammation. **(C)** Polyamines (e.g., spermidine and putrescine) and aromatic amino acid (AAA)–derived metabolites (e.g., kynurenine derivatives) control mitochondrial function, inflammation and chromatin remodeling. A mode of action promotes hypusination of eukaryotic initiation factor 5A (eIF5A), which regulates protein translation and autophagy, notably mitochondrial biogenesis and mitophagy. Spermidine can also increase acetylhypusination of receptor-interacting protein kinase 1 (RIPK1) to suppress its activity and improve insulin sensitivity. N-acetyltransferase 1 (NAT1) deficiency decreases spermidine levels, which consequently increases RIPK1 activity, contributing to increased inflammation and insulin resistance. AI-assisted figure created with FigureLabs.

Short-chain fatty acids (SCFAs) play an important role in metabolic regulation and have been extensively studied (Figure 1A; Table 1). SCFAs, primarily acetate, propionate and butyrate, are generated through anaerobic fermentation of dietary fibers and non-digestible polysaccharides by gut bacteria such as *Akkermansia muciniphila*, *Faecalibacterium prausnitzii* and other members of the Firmicutes, Actinobacteria and Bacteroidetes phyla (Van der Hee and Wells, 2021). SCFAs contribute to host physiology by acting as energy substrates and by mediating signaling and epigenetic effects.

**Table 1:** Summary of recent studies demonstrating circulating concentrations of selected short-chain fatty acids: acetate, propionate and butyrate.

Study description and reference	Acetate	Propionate	Butyrate
Relationship between serum SCFA concentrations and body composition in T2D (*n* = 430) (Hsu et al., 2025)	93.9 versus 93.0 μm in participants with lower versus higher lean mass (*p* = 0.84). 97.2 versus 89.1 μm in participants with lower versus higher fat mass (*p* = 0.08).	15.6 versus 15.4 μm in participants with lower versus higher lean mass (*p* = 0.84). 16.5 versus 15.3 μm in participants with lower versus higher fat mass (*p* = 0.48).	8.1 versus 1.2 μm in participants with lower versus higher lean mass (*p* = 0.76). 8.1 versus 8.1 μm in participants with lower versus higher fat mass (*p* = 0.39).
Associations of circulating SCFA concentrations with specific food consumption patterns and prospectively assess association with T2D development in at-risk subjects (*n* = 5,072 baselines; *n* = 2,408 follow-up) (Llaurado et al., 2025)	Baseline concentrations of 27.3, 28.0 and 31.0 μmoL/L, increasing trend with increasing consumption of high-fiber food (*p* < 0.0001). No association with T2D at baseline. No association with developing T2D at follow-up.	Baseline concentrations of 3.3, 3.4 and 3.6 μmoL/L, increasing trend with increasing consumption of high-fiber food (*p* < 0.0001). Associated with T2D at baseline, with higher serum concentrations being in particpants with T2D (*p* > 0.001). No association with developing T2D at follow-up.	Baseline concentrations of 11.0, 11.5 and 11.9 μmoL/L, increasing trend with increasing consumption of high-fiber food (*p* < 0.0001). No association with T2D at baseline. Associated with developing T2D at follow-up, higher serum concentrations in those that develop T2D (*p* = 0.04)
Association of branched SCFAs (BSCFAs) metabolism, measured in plasma alongside other SCFAs, with dysglycemia and oral glucose tolerance test results in the miles cohort (*n* = 345). A subset of participants with high BSCFAS concentrations (H-BSCFA) was compared to the rest of the population (L-BSCFA) (Aslamy et al., 2024; Jensen et al., 2020)	6237.1 versus 2335.8 ng/mL in H- versus L-BSCFA (*p* < 0.0001).	277.7 versus 76.7 ng/mL in H- versus L-BSCFA (*p* < 0.0001).	38.1 versus 28.2 ng/mL in H- versus L-BSCFA (*p* = 0.0027).
	Age, BMI, waist circumference and sex distribution were comparable between h- versus L-BSCFA groups. Of glycemic traits, fasting glucose, glucose at 120 min, glucose auc, fasting C-peptide, and C-peptide auc and insluin clearance were all higher in the L-BSCFA group compared to the H-BSCFA group. Disposition index was lower in the L-BSCFA group compared to the H-BSCFA group.		

SCFA: short-chain fatty acids; T2D: type 2 diabetes; BSCFA: branched short-chain fatty acids; BMI: body mass index.

SCFAs, particularly butyrate and propionate, exert beneficial metabolic effects by enhancing glucose uptake, improving insulin sensitivity and reducing inflammation (Das et al., 2021). However, the metabolic effects of SCFAs are not uniformly beneficial and may depend on metabolite concentration, tissue context, host metabolic status and microbial composition. Furthermore, findings from experimental models have not always translated consistently to human studies, highlighting the complexity of SCFA-mediated regulation. Butyrate has the strongest effect, as demonstrated by its improving fasting blood glucose and insulin sensitivity in models of diabetes (Zhang et al., 2024). Importantly, the associated chromatin effects can be microbiota-independent, as SCFA supplementation in germ-free mice recapitulates microbiota-driven chromatin changes and transcriptional responses (Krautkramer et al., 2016). These findings highlight that epigenetic responses attributed to microbial metabolites are not exclusively microbiota dependent. Rather, microbial products interact with other metabolic and environmental cues to regulate chromatin accessibility and gene expression. Mechanistically, butyrate promotes glucose homeostasis and protects cells by modulating the p38/MAPK and apoptotic signaling pathways. In addition, it functions as a histone deacetylase (HDAC) inhibitor. HDAC inhibition increases histone acetylation, relaxes chromatin structure and enables reactivation of silenced genes (Khan and Jena, 2014). Propionate also acts as an HDAC inhibitor, altering chromatin accessibility and transcriptional outcomes (Krautkramer et al., 2016) (Figure 1A).

### 2.2 Beyond SCFAs: emerging bacterial metabolites

While SCFAs are well-known for their influence, growing evidence also highlights less-studied microbial metabolites, including tryptophan-derived indoles, polyamines and AAA derivatives.

#### 2.2.1 Tryptophan-derived indole metabolites

One of the most promising metabolites is indole-3-propionic acid (IPA), a tryptophan-derived compound produced by gut bacteria, mainly *Clostridium* species (Xiao et al., 2020; Zhao et al., 2019) (Figure 1B). IPA is absorbed by epithelial cells and enters the circulation to reach multiple target tissues, including the liver (Wikoff et al., 2008). Higher circulating IPA concentrations are associated with lower risk of obesity, T2D (de Mello et al., 2017), MASLD (Min et al., 2024) and cardiovascular disease (CVD) (Xue et al., 2022). Evidence from the Finnish Diabetes Prevention Study further demonstrated that elevated serum IPA concentrations predicted a lower incidence of T2D and were associated with better *β*-cell function, improved insulin sensitivity and reduced low-grade inflammation (de Mello et al., 2017). Conversely, individuals with T2D consistently have lower circulating IPA levels (Sehgal et al., 2021).

Mechanistic studies demonstrate that IPA exerts pleiotropic effects. It acts as a ligand for the aryl hydrocarbon receptor (AHR), influencing immune and inflammatory pathways (Laurans et al., 2018; Li et al., 2022). At the epigenetic level, IPA enhances histone acetylation and increases the expression of genes such as SIRT1 and FOXO3, which maintain mitochondrial function and cellular stress resistance (Sehgal et al., 2021). Moreover, serum IPA has been associated with DNA methylation levels in the liver, and its dietary supplementation suppresses expression of the methyltransferase NNMT in the heart. These properties reflecting DNA as well as protein methylation are reported for targets that regulate oxidative stress, inflammation and glucose metabolism (Ilha et al., 2025; Wang et al., 2024). Recent findings show that IPA enhances S-adenosylmethionine (SAM) synthesis through activation of methionine adenosyltransferase 2A (MAT2A). Increased SAM availability promotes DNA methylation of Usp16, resulting in TLR4 degradation and suppression of NF-κB signaling (Han et al., 2025). Recent studies further suggest that IPA regulates hepatic fibrogenesis through coordinated epigenetic and metabolic mechanisms (Ilha et al., 2025). These effects promote hepatic stellate cell inactivation while reducing oxidative stress and inflammatory signaling, thereby limiting MASLD progression (Ilha et al., 2025). Beyond metabolism, IPA also modulates apoptosis and fibrosis-related genes (Ilha et al., 2025), reducing neuroinflammation, by inhibiting the RAGE-JAK2-STAT3 pathway (Hao et al., 2025).

Collectively, IPA is a promising microbiota-derived metabolite with epigenetic, metabolic and anti-inflammatory benefits. Nevertheless, much of the mechanistic evidence derives from experimental studies, and the extent to which these findings translate into clinically meaningful outcomes in T2D remains uncertain. In addition, circulating IPA concentrations are influenced by diet, microbiome composition and host metabolism, complicating interpretation of causal relationships.

#### 2.2.2 Polyamines

Polyamines, include spermidine, putrescine and cadaverine, also have emerging significance (Figure 1C). Fecal analyses indicate that putrescine is the most abundant in the colon, followed by spermine, spermidine and cadaverine (Matsumoto and Benno, 2007). Lower circulating spermidine concentrations are associated with T2D, suggesting a metabolically protective role, although excessively high levels may not provide additional benefits (Zhang et al., 2024). These observations suggest that the relationship between spermidine and metabolic health may not be linear, and the optimal range required for beneficial effects remains unclear. Aging also shows a progressive decline in spermidine levels in humans and animals, potentially contributing to age-related metabolic dysfunction (Hofer et al., 2021).

At the organ level, spermidine plays a central role in maintaining intestinal homeostasis and metabolic balance (Zhang et al., 2024). It influences systemic metabolic pathways, including the Krebs cycle, vitamin digestion and absorption and insulin signaling, highlighting broad regulatory capacity (Jiang et al., 2023).

In addition, spermidine influences chromatin remodeling by inhibiting histone acetyltransferase (HAT) activity, leading to histone hypoacetylation and the induction of autophagy, thereby promoting cellular homeostasis and longevity (Eisenberg et al., 2009; Satarker et al., 2024). These chromatin-dependent effects further support the role of spermidine in metabolic and epigenetic regulation (Satarker et al., 2024). These mechanisms have been comprehensively reviewed elsewhere (Satarker et al., 2024).

At cellular and subcellular levels, spermidine exerts epigenetic and post-translational regulatory effects. It promotes hypusination of eukaryotic initiation factor 5A (eIF5A), a modification essential for protein translation and regulating autophagy, thereby influencing mitochondrial biogenesis and mitophagy (Hofer et al., 2021). Spermidine also induces acetylhypusination of receptor-interacting protein kinase 1 (RIPK1), suppressing its activation and improving insulin sensitivity (Zhang et al., 2024). Importantly, N-acetyltransferase 1 (NAT1) deficiency reduces spermidine levels, leading to increased RIPK1 activity, inflammation and insulin resistance. These adverse metabolic effects can be reversed by spermidine supplementation or RIPK1 inhibition in models of diabetes (Zhang et al., 2024), further supporting its mechanistic relevance in metabolic disease.

#### 2.2.3 Aromatic amino acid (AAA)-derivatives

The gut microbiome also metabolizes AAAs such as tryptophan, phenylalanine and tyrosine into a range of compounds that accumulate in the circulation and act systemically (Dodd et al., 2017). Within the kynurenine pathway, tryptophan metabolism yields kynurenine, 3-hydroxykynurenine and 3-hydroxyanthranilic acid, each of which has been linked to metabolic regulation (Su et al., 2022). Indole derivatives of tryptophan metabolism are particularly relevant, as they contribute to development of metabolic syndrome and obesity upon high-fat feeding (Zhu et al., 2024). Recent studies further demonstrate that AAA-derived metabolites regulate immune and metabolic homeostasis through receptor-mediated signaling, including activation of the AHR, thereby influencing intestinal barrier integrity, inflammatory responses and glucose metabolism (Shin and Bang, 2025; Zhang et al., 2026). Emerging evidence also indicates that host–microbiota metabolic interactions involving AAA metabolites influence chromatin remodeling and transcriptional regulation, linking microbial metabolism to epigenetic control of immune and metabolic pathways (Zhang et al., 2026). Although precise epigenetic mechanisms remain to be fully defined, accumulating evidence suggests that AAA derivatives influence chromatin remodeling and metabolic pathways (McCall et al., 2009; Krautkramer et al., 2017).

These reports demonstrate that bacterial metabolites can indeed influence T2D by reshaping the host epigenome (Figure 1C). The most prominent mechanisms include HDAC inhibition by SCFAs, histone acetylation and DNA methylation mediated by IPA, and hypusination or other post-translational modifications regulated by spermidine. By altering chromatin accessibility, transcriptional activity and inflammatory signaling, these metabolites establish a direct mechanistic link between gut microbiota and host metabolic health.

## 3 Tissue- and cell-specific epigenetic effects of microbial metabolites

**Figure 2:**
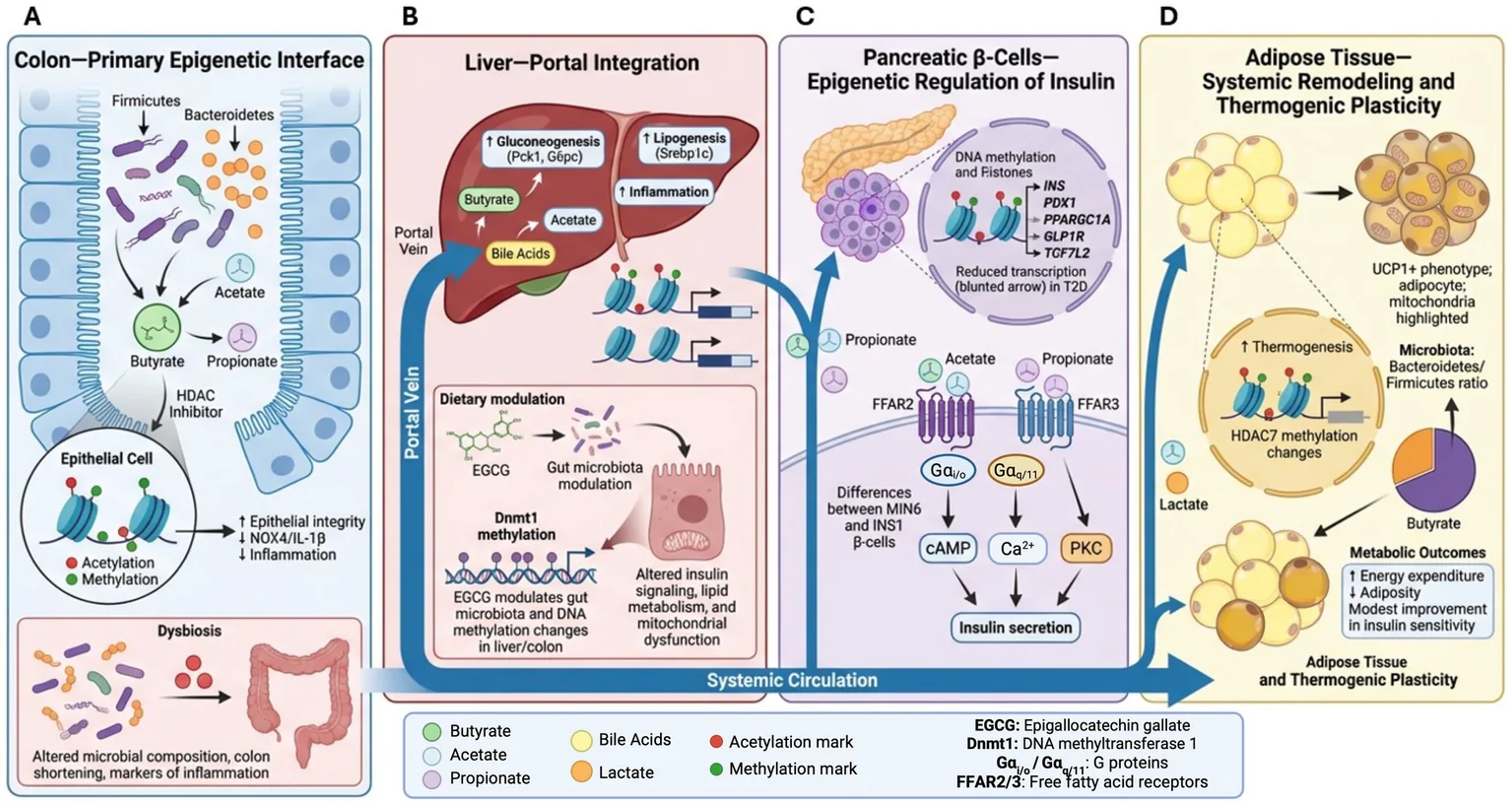
Tissue- and cell-specific epigenetic effects of gut microbiota-derived metabolites on metabolic regulation across the gut, liver, pancreas and adipose tissue. **(A)** The colon is the primary epigenetic interface for microbial metabolites. Fermentation by dominant taxa such as *Firmicutes* and *Bacteroidetes* generates short-chain fatty acids (SCFAs), including acetate, propionate, and butyrate. Butyrate acts as a histone deacetylase (HDAC) inhibitor in epithelial cells, promoting or maintaining histone acetylation, strengthening epithelial integrity, and suppressing inflammatory signaling. Dysbiosis alters this balance and is associated with shortened colon architecture and increased inflammatory markers, such as NADPH oxidase 4 (NOX4) and Interleukin-1*β* (IL-1*β*). **(B)** In the liver, portal delivery of SCFAs and bile acids links intestinal microbial metabolism to hepatic gene regulation. Through target genes and proteins, including Phosphoenolpyruvate carboxykinase 1 (Pck1), Glucose-6-phosphatase (G6pc) and Sterol regulatory element-binding protein 1c (Srebp1c), metabolites contribute to epigenetic control of pathways governing gluconeogenesis, lipogenesis, and inflammation. These interactions influence hepatic insulin sensitivity and systemic glucose homeostasis. Dietary modulation of microbiota, for example by catechins like Epigallocatechin-3-gallate (EGCG), also shapes metabolite composition and downstream epigenetic effects. DNA methylation is enhanced for DNA methyltransferase 1 (Dnmt1) upon dietary modulation of microbiota by EGCG, and this occurs in both the liver and the colon. **(C)** In pancreatic *β*-cells, acetate and propionate signal through the free fatty acid receptors 2 (FFAR2) and 3 (FFAR3), activating intracellular pathways involving cyclic AMP (cAMP) Ca^2+^ and Protein kinase C (PKC). These signaling cascades regulate transcription of genes associated with insulin secretion and *β*-cell identity, including Insulin (INS), Pancreatic and duodenal homeobox 1 (PDX1), PPARG coactivator 1 alpha (PPARGC1A), Glucagon like peptide 1 receptor (GLP1R) and Transcription factor 7-like 2 (TCF7L2). Altered DNA methylation and altered transcription of these target genes are observed in type 2 diabetes. **(D)** In adipose tissue, microbial metabolites can promote thermogenic remodeling, through methylation and through HDAC-activity, notably HDAC7. Gut microbiota, notably a balance between *Bacteroidetes* and *Firmicutes*, will influence metabolites in circulation, and this balance also affects adipose thermogenic capacity. White adipocytes acquiring expression of Uncoupling protein 1 (UCP1) have a more appropriate adaptation to metabolic stress and increased thermogenesis. AI-assisted figure created with FigureLabs.

Microbiota-derived metabolites reach tissues in a defined anatomical sequence: originating in the colon and entering the portal circulation before systemic distribution, their epigenetic effects exhibit tissue-specific patterns (Figure 2). Dysbiosis associated with obesity and T2D alters metabolite availability and thereby reshapes chromatin remodeling across metabolic organs (Miro-Blanch and Yanes, 2019; Krautkramer et al., 2017).

### 3.1 Colon: the primary epigenetic Interface

The colon is the first site exposed to high concentrations of microbial metabolites and is the principal interface for host epigenetic interactions (Figure 2A). Microbial colonization profoundly alters global histone acetylation and methylation in colonic epithelial cells (Krautkramer et al., 2016). SCFAs, particularly butyrate, function as HDAC inhibitors and can also contribute to acetyl-CoA pools, directly influencing chromatin accessibility and gene transcription.

These epigenetic changes regulate epithelial barrier integrity, oxidative stress responses and inflammatory signaling. In the MKR murine model of diabetes, dysbiosis was associated with colon shortening and increased inflammatory markers, including NOX4 and IL-1*β*. Butyrate supplementation partially restored homeostatic gene expression, supporting a causal role in chromatin remodeling in the colon (Noureldein et al., 2020). Thus, the colon acts as an active metabolic and epigenetic sensor, translating luminal microbial signals to the host.

### 3.2 Liver: portal integration of microbial signals

Following absorption, microbial metabolites enter the portal vein and reach the liver, making it the first systemic organ to be exposed (Figure 2B). In T2D, hepatic dysfunction involves insulin resistance, increased gluconeogenesis, enhanced lipogenesis, mitochondrial impairment and inflammation (Alabdulaali et al., 2023; Niranjan et al., 2023).

These features are accompanied by epigenetic alterations at promoters and enhancers of genes regulating key metabolic pathways. These include genes regulating gluconeogenesis (*PECK11*, *G6PC*), lipogenesis (*SREBP1C*) and inflammation. Changes in histone acetylation and DNA methylation at these loci contribute to transcriptional reprogramming of the liver’s metabolic networks (Yu et al., 2021; Ramatchandirin et al., 2023). In addition, microbiota-dependent chromatin remodeling in hepatocytes correlates with altered insulin signaling and lipid metabolism (Krautkramer et al., 2016).

Dietary modulation of microbiota further influences hepatic epigenetics. For example, epigallocatechin-3-gallate (EGCG) alters gut microbiota composition and reshapes DNA methylation in liver and colon, in a tissue-specific manner, upon diet-induced obesity (Liu et al., 2021; Remely et al., 2017). *Dnmt1* methylation was however enhanced across both tissues, with notable alterations detected at distinct CpG sites (Remely et al., 2017).

Collectively, these studies position the liver as a central integrator of microbiota-derived epigenetic signals affecting systemic glucose and lipid homeostasis.

### 3.3 Pancreatic *β*-cells: epigenetic regulation of insulin production

After hepatic processing, microbial metabolites can influence pancreatic islets (Figure 2C). In *β*-cells, epigenetic regulation is essential for developmental programming and maintenance of insulin secretion.

During development, DNA methylation and histone modifications establish transcriptional networks defining *β*-cell identity and insulin expression. Disrupting these mechanisms impairs insulin transcription and contributes to diabetes pathogenesis (Kaimala et al., 2022).

As HDAC inhibitors, SCFAs also modulate chromatin accessibility in *β*-cells, such regulation may affect key transcription factors required for insulin expression and glucose responsiveness (Wang et al., 2026; Sun et al., 2021).

Studies in human pancreatic islets from donors with T2D provide evidence of epigenetic dysregulation. This involved altered DNA methylation in INS, PDX1, PPARGC1A (encoding PGC-1*α*) and GLP1R, genes essential for insulin production and *β*-cell identity (Volkmar et al., 2019). These methylation changes were associated with impaired transcriptional activity. Additional work identified differential methylation in PDX1 and TCF7L2, both central regulators of *β*-cell development, function and diabetes susceptibility (Sommese et al., 2018; Chakarova et al., 2019).

SCFAs can also signal through G protein–coupled receptors. In 2003, GPR41 and GPR43 were deorphanized and renamed free fatty acid receptors 2 and 3 (FFAR2/3) (Brown et al., 2003). Upon ligand binding, FFAR2 can couple either to pertussis toxin–sensitive G*α*_i/o_ proteins or to G*α*_q/11_ proteins, leading to modulation of intracellular cyclic AMP (cAMP) levels or activation of calcium/PKC signaling pathways (Priyadarshini et al., 2016). These influence *β*-cell stimulus–secretion coupling, with context-dependent physiological relevance.

Notably, G*α*_q/11_ coupling was reported predominantly in MIN6 *β*-cells, whereas G*α*_i/o_ signaling predominates in INS1 cells (McNelis et al., 2015), suggesting species or responsiveness specificity. Acetate and propionate are the most potent FFAR2 agonists (EC₅₀ ≈ 20–300 μM). However, circulating propionate concentrations *in vivo* are typically below 20 μM, raising questions regarding receptor activation thresholds under physiological conditions (Brusselle et al., 2013).

### 3.4 Adipose tissue: systemic epigenetic remodeling and thermogenic plasticity

Following systemic distribution, microbial metabolites reach adipose tissue, where they influence inflammation, lipid handling and thermogenic remodeling (Figure 2D).

With insulin resistance, adipose tissue undergoes hypertrophy, hypoxia, immune-cell infiltration and extracellular matrix remodeling, processes that drive chronic low-grade inflammation, lipolysis and systemic metabolic dysfunction (Lafontan, 2014; Kawai et al., 2021; Zhao and Yue, 2025). Epigenetically, microbiota composition is associated with differential DNA methylation in adipose tissue, affecting chromatin structure through the activity of HDACs, notably HDAC7 (Ramos-Molina et al., 2019).

A major adaptive response to metabolic stress is thermogenic remodeling, often described as white-to-beige (or brown-like) transition (Kurylowicz and Puzianowska-Kuznicka, 2020; Yin et al., 2022). During this process, subsets of white adipocyte acquire a UCP1-expressing oxidative phenotype. This phenotype is characterized by enhanced mitochondrial biogenesis, uncoupled respiration and increased lipid and glucose oxidation, elevating energy expenditure (Oliveira et al., 2016; Bettini et al., 2019). Although beiging is associated with metabolic improvement, its induction does not necessarily translate into proportional improvements in systemic insulin sensitivity (Cheng et al., 2021). Improvements in glucose homeostasis instead arises from increased thermogenesis or reduced adiposity rather than from insulin sensitization (Xiao et al., 2015; BonDurant et al., 2017; Laeger et al., 2017).

Gut microbiota contributes to thermogenic programming (Riedl et al., 2021). Microbiota depletion or remodeling alter SCFA levels and promote adipocyte beiging, accompanied by systemic metabolic changes (Colangeli et al., 2023; Li et al., 2017; Sahuri-Arisoylu et al., 2016).

Variation in the Bacteroidetes-to-Firmicutes ratio has also been associated with differential DNA methylation patterns in adipose tissue, including altered methylation of HDAC7 (Ramos-Molina et al., 2019). Experimental models demonstrate that microbial colonization and diet can reshape histone acetylation and methylation patterns across metabolic tissues, including white adipose tissue (Krautkramer et al., 2016). Because many butyrate-producing taxa belong to the Firmicutes phylum, shifts in the Bacteroidetes/Firmicutes balance may reflect differences in SCFA–producing capacity, although butyrate synthesis is not restricted exclusively to Firmicutes lineages (Vital et al., 2014).

## 4 Intergenerational epigenetic programming of metabolism

The epigenome is a mediator of intergenerational transmission of metabolic risk; such can be explained, at least in part, by an axis that connects parental nutrition to the microbiome and its products, and their epigenetic manifestations (Yang et al., 2025). Maternal nutritional status in particular influences the composition and metabolic activity of the gut microbiota, thereby altering the production of microbiota-derived metabolites such as short-chain fatty acids, vitamins, and one-carbon metabolites that can regulate DNA methylation and histone modification during fetal development and early postnatal life. The microbiome thus functions as a mechanistic intermediate between maternal diet and environmental exposures and offspring metabolic programming. Experimental and human studies indicate that these pathways can influence obesity, insulin resistance, and other cardiometabolic traits in offspring, supporting biological relevance of microbial metabolites in developmental programming.

### 4.1 Maternal diet as a driver of offspring epigenetic programming

**Figure 3:**
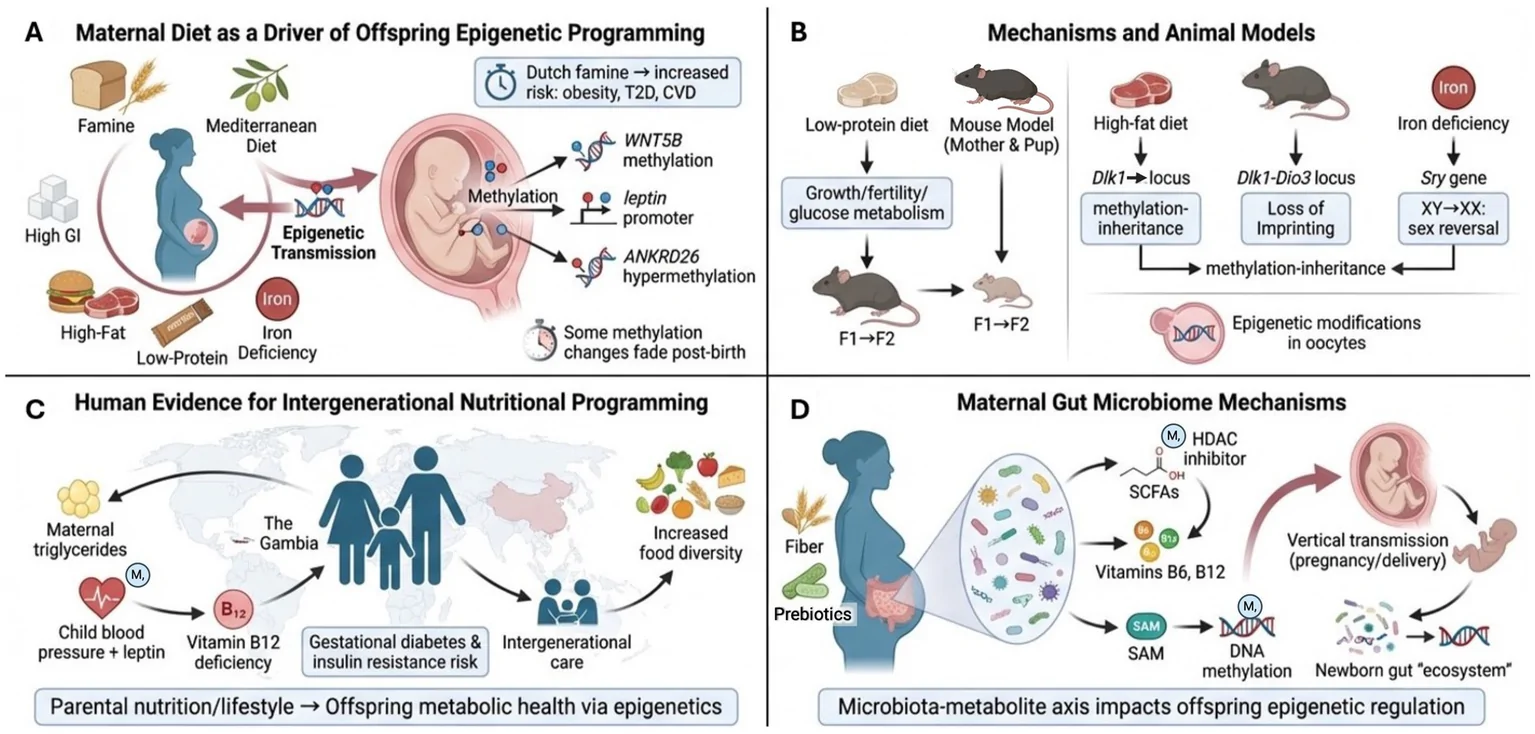
Intergenerational epigenetic programming of offspring metabolism driven by maternal diet, environmental exposures and the gut microbiome. **(A)** Maternal diet during pregnancy, including high-fat, low-protein, high glycemic index (GI) diets, and micronutrient deficiencies, can alter fetal DNA methylation altering gene expression, with effects on loci such as Wnt Family Member 5B (WNT5B), leptin, and Ankyrin repeat domain-containing protein 26 (ANKRD26). These changes may persist beyond birth and contribute to offspring metabolic disease risk, including type 2 diabetes (T2D) and cardiovascular disease (CVD), although some epigenetic marks may be partially reversible after delivery. **(B)** In animal models, maternal dietary perturbations alter offspring growth, fertility, glucose metabolism, and genomic imprinting, including at the Delta-like non-canonical Notch ligand 1 (Dlk1)-Iodothyronine Deiodinase 3 (Dio3) loci and at the Sex-determining region Y protein (Sry) gene. These studies support existence of transgenerational epigenetic inheritance with evidence that some effects can be transmitted across generations (F1 to F2). These epigenetics modifications take place at the oocyte stage. **(C)** In humans, maternal nutritional status and micronutrient availability, including vitamin B12 status, are associated with offspring cardiometabolic outcomes such as insulin resistance, blood pressure, and metabolic risk. The maternal metabolic environment can therefore shape offspring health through epigenetic pathways across developmental windows. **(D)** The maternal gut microbiome, shaped by diet, fiber, and prebiotic intake, produces metabolites such as short-chain fatty acids (SCFAs), vitamins, and S-adenosylmethionine (SAM) precursors that regulate epigenetic pathways including DNA methylation and histone deacetylase (HDAC) activity. Maternal microbial communities can also be transferred vertically during pregnancy and delivery, contributing to early-life metabolic programming in offspring. AI-assisted figure created with FigureLabs.

The influence of maternal nutritional status on offspring health through epigenetic mechanisms was first illustrated by observations from the Dutch famine of 1944–1945 (Veenendaal et al., 2013). Individuals exposed to famine *in utero* had increased risk of obesity, T2D and CVD in adulthood (Figure 3A). These observations provided foundational support for the Developmental Origins of Health and Disease (DOHaD) (Veenendaal et al., 2013) hypothesis, that early-life nutritional adversity can induce long-lasting epigenetic alterations detectable decades later.

Subsequent studies expanded this, although the strength, persistence and tissue specificity of epigenetic changes vary. For example, maternal adherence to Mediterranean diet during pregnancy was associated with differential DNA methylation in newborn cord blood (Sasaki et al., 2023; Kupers et al., 2022) (Figure 3A). Among identified loci was WNT5B, a member of the WNT signaling family known for roles in embryonic development, cell differentiation and proliferation, and in adipogenesis and metabolic regulation (Bagchi and MacDougald, 2021). These methylation differences were not detectable in later childhood, raising questions about stability and long-term functional relevance of diet-induced neonatal epigenetics (Kupers et al., 2022).

Similarly, randomized trials of low-glycemic index (GI) dietary interventions during pregnancy have demonstrated modest but statistically significant DNA methylation changes in cord blood. These changes involved genes associated with cardiac development and immune regulation. These changes were independent of maternal BMI and birth weight, suggesting that dietary quality itself may influence the fetal epigenome beyond effects mediated by maternal adiposity (Geraghty et al., 2020).

In pregnancies complicated by gestational diabetes mellitus (GDM), differential DNA methylation has been reported in cord blood and peripheral blood mononuclear cells, particularly at CpG sites associated with transcriptional regulation and cell signaling. Importantly, maternal lifestyle interventions, including structured low-GI diets and physical activity, attenuated some of these epigenetic alterations (Antoun et al., 2020). These findings suggest that adverse intrauterine metabolic exposure may be modified, reinforcing the concept that maternal metabolic status shapes offspring epigenetic trajectories during critical developmental windows.

### 4.2 Models revealing mechanisms of transgenerational nutritional programming

Animal studies provide deeper mechanistic insights into transgenerational diet effects. A maternal low-protein diet during lactation disrupted offspring growth, fertility and glucose metabolism across generations, with epigenetic modifications in oocytes pointing to an inheritance mechanism (Gu et al., 2024). Similarly, maternal obesity in mice produced more severe reproductive and metabolic defects in offspring than paternal obesity, including impaired ovarian function and altered mitochondrial and inflammatory pathways (Ramadan et al., 2023). High-fat maternal diets have also been shown to disrupt genomic imprinting at the Dlk1-Dio3 locus, leading to sustained loss of imprinting and DNA methylation changes that persist for at least two generations, linking maternal nutrition to heritable metabolic dysfunction (Van de Pette et al., 2022). Further evidence shows that maternal iron deficiency during pregnancy can alter embryonic epigenetic regulation by impairing activation of the Sry gene through disruption of the iron-dependent histone demethylase KDM3A, resulting in male-to-female sex reversal in a subset of mouse embryos (Okashita et al., 2025). These studies highlight how epigenetic mechanisms transmit maternal nutritional status to offspring, altering gene regulation, development and long-term metabolic health (Figure 3B).

### 4.3 Human evidence for intergenerational nutritional programming

In the DOHaD context, epidemiological observations indicate that nutritional status and lifestyle factors before and during pregnancy influence developmental programming that affects offspring metabolic health (Fleming et al., 2018) (Figure 3C). For example, research conducted in The Gambia demonstrated that maternal and paternal environmental exposures, including nutrient restriction and maternal stress, significantly affected offspring growth and psychosocial development, highlighting that parental nutrition and environmental conditions shape long-term health outcomes in resource-limited settings (Bajinka et al., 2022).

Similarly, a study examining intergenerational care behavior and nutritional intake among rural residents in China, using data from the China Family Panel Studies (2010–2014), reported that households with intergenerational care exhibited higher food diversity, with approximately a 22.4% increase in consumption, likely through increased labor participation and income among young mothers, which improved household dietary structure (Bei et al., 2018; Romieu et al., 2017).

Further evidence from cohort studies indicates that maternal metabolic status during pregnancy influences offspring cardiometabolic outcomes. Elevated maternal triglyceride levels during gestation are associated with higher blood pressure and increased circulating leptin in children, accompanied by reduced DNA methylation at the leptin promoter in offspring blood cells, suggesting that maternal lipid metabolism may induce persistent epigenetic modifications that influence long-term metabolic risk in offspring (Lin et al., 2021). Consistently, human epigenetic studies show that obesity is associated with hypermethylation of metabolic regulatory genes such as ANKRD26, which correlates with increased BMI, inflammatory status and cardiometabolic risk factors (Desiderio et al., 2019). Moreover, maternal vitamin B12 deficiency during pregnancy is linked to gestational diabetes and metabolic alterations in offspring, including increased susceptibility to insulin resistance (Dunphy and Tang, 2023; He et al., 2022).

Collectively, these studies highlight how parental nutritional and metabolic conditions can influence offspring health through mechanisms involving long-lasting epigenetic modifications, providing understanding of intergenerational risk transmission of metabolic disease.

### 4.4 Maternal gut microbiome in intergenerational epigenetic programming

Maternal gut microbiota has emerged as an important mediator of intergenerational epigenetic programming. Maternal diet can shape composition, diversity and metabolic activity of gut microbiota, thereby altering production of microbiota-derived metabolites with epigenetic regulatory potential (Yang et al., 2025) (Figure 3D). SCFAs function as HDAC inhibitors and bacterially synthesized vitamins, such as B₆ and B₁₂, contribute to one-carbon metabolism and the generation of S-adenosylmethionine (SAM), the universal methyl donor required for DNA and histone methylation (Li, 2018).

Microbial signals also influence host microRNA expression, pointing to a microbiota–miRNA axis in gene regulation (Lin et al., 2024). Importantly, maternal microbial communities also influence offspring through vertical transmission during pregnancy, delivery and early-life colonization, shaping the initial microbial ecosystem that interacts with host epigenetic regulation. Together, these mechanisms suggest that maternal microbiota composition is a key determinant of epigenetic programming in offspring, adding a new mechanistic dimension to the DOHaD framework.

Collectively, human and preclinical studies converge on the idea that maternal diet exerts profound epigenetic effects on offspring health (Li, 2018). While some effects, such as those associated with famine exposure or high-fat diets, persist across generations, others are transient and context-dependent. The gut microbiome provides a mechanistic bridge linking maternal nutrition, microbial metabolism, epigenetic regulation and offspring health outcomes, highlighting new opportunities for microbiome-targeted interventions aimed at managing intergenerational transmission of metabolic disease (Figure 3D).

## 5 Therapeutic innovations targeting the microbiota–epigenetic axis

Emerging knowledge on the interplay between gut microbiota, metabolites and epigenetic regulation has opened new avenues for therapeutic innovation in diabetes. Importantly, current standard-of-care remains centered on pharmacological glucose-lowering therapies, including metformin, GLP-1 receptor agonists, SGLT2 inhibitors and insulin (American Diabetes Association Professional Practice C, 2024). Microbiota- or epigenetics-based interventions, supported by mechanistically relevant translational or clinical studies cited in this review (Table 2), are not substitutes for these treatments but are being explored as complementary or future therapeutic strategies. Although accumulating mechanistic evidence supports a role for microbiota-derived metabolites in regulating epigenetic pathways relevant to T2D, clinical evidence remains limited and heterogeneous. Most available data originate from experimental models, and relatively few studies have been conducted in humans. Furthermore, no advanced-phase clinical trials have specifically proven the clinical benefit of metabolite-mediated epigenetic mechanisms. Consequently, microbiota-based interventions should currently be regarded as complementary rather than substitutive approaches alongside established therapies.

**Table 2:** Summary of human clinical evidence for nutritional and epigenetic programming of metabolic health.

Study/Population	Study design	Intervention/Exposure	Main findings	Epigenetic evidence	Clinical relevance	Reference
Adults with T2D	Cross-sectional	Serum spermidine	Non-linear association between serum spermidine and T2D; physiological levels associated with lower T2D risk	Proposed mechanisms include eIF5A hypusination and autophagy (not directly assessed)	Supports spermidine as a potential biomarker for metabolic health.	(Zhang et al, 2024)
Individuals with obesity	Cross-sectional	Gut microbiota composition	Gut microbiota was associated with adipose DNA methylation, including HDAC7 and IGF2BP2	Differential adipose DNA methylation	Links gut microbiota with adipose epigenetic regulation.	(Ramos-Molina et al, 2019)
Pregnant women and offspring	Prospective cohort	Maternal Mediterranean diet adherence	Higher adherence was associated with beneficial neonatal microbiota and differential DNA methylation.	Differential DNA methylation in cord blood and placenta	Supports maternal diet in shaping offspring microbiota and epigenetic programming	(Sasaki et al, 2023)
Mother–child pairs (PACE Consortium)	Meta-analysis	Maternal Mediterranean diet	Higher adherence was associated with increased WNT5B DNA methylation in cord blood	Differential cord blood DNA methylation	Suggests maternal diet may influence neonatal epigenetic programming	(Kupers et al, 2022)
Pregnant women (ROLO Study)	Randomized dietary intervention	Low-glycaemic index diet	Limited DNA methylation differences in offspring at 5 years.	Differential methylation in insulin- and metabolism-related pathways	Maternal diet may influence the offspring epigenome.	(Geraghty et al, 2020)
Women with gestational diabetes mellitus	Randomized lifestyle intervention	Low-GI diet and physical activity	Lifestyle intervention attenuated offspring DNA methylation changes.	Differential CpG methylation in cord blood.	Supports reversibility of fetal epigenetic alterations.	(Antoun et al, 2020)
Gambian population	Cross-sectional	Parental nutrition and environmental exposures	Parental nutrition and environmental exposures were associated with offspring growth and psychosocial development.	Indirect evidence of developmental programming.	Supports the DOHaD hypothesis in humans	(Bajinka et al, 2022)
Pregnant women and offspring	Cohort study	Elevated maternal triglycerides	Higher offspring blood pressure and leptin levels.	Reduced leptin promoter DNA methylation.	Maternal hypertriglyceridaemia may program offspring cardiometabolic risk.	(Lin et al, 2021)
Lean and obese adults	Cross-sectional	Obesity	Obesity was associated with reduced ANKRD26 expression and increased inflammation	ANKRD26 promoter hypermethylation.	Identifies ANKRD26 methylation as a potential biomarker of cardiometabolic risk.	(Desiderio et al, 2019)
Pregnant women	Systematic review and meta-analysis	Maternal vitamin B12 deficiency	Associated with increased GDM risk.	One-carbon metabolism and DNA methylation	Highlights the importance of maternal vitamin B12 status.	(He et al, 2022)

T2D: Type 2 diabetes; eIF5A: Eukaryotic translation initiation factor 5A-1; HDAC7: Histone deacetylase 7; IGF2BP2: Insulin-like growth factor 2 mRNA-binding protein 2; WNT5B: Wnt Family Member 5B; DOHaD: Developmental origins of the health and disease; ANKRD26: Ankyrin repeat domain-containing protein 26; GDM: Gestational diabetes mellitus.

Epigenetic modification represents an active and rapidly evolving area of biomedical research. Multiple epigenetic-targeting drugs, such as HDAC inhibitors (Daniel et al., 2015) and DNA methyltransferase (DNMT) inhibitors (Sato et al., 2017), have already been clinically implemented in oncology, providing proof-of-concept that epigenetic pathways are pharmacologically tractable (Majchrzak-Celinska et al., 2021; Patnaik and Anupriya., 2019; de Lera and Ganesan, 2016). Although their application in metabolic diseases remains in earlier stages, cancer research offers a well-established framework to inform development of epigenetic therapies for diabetes (Sarno et al., 2021; Napoli et al., 2020).

In parallel, pro-, pre- and post-biotic strategies are gaining attention for their tolerability, safety profiles and adaptability (Kandari et al., 2024). By modulating microbial composition or delivering specific metabolites with epigenetic activity, these approaches aim to reshape host chromatin landscapes and metabolic signaling networks. While still under investigation, such strategies represent a promising adjunctive direction.

### 5.1 Dietary modulation of microbial–epigenetic signaling

Dietary modification represents a primary and clinically accessible strategy for shaping gut microbiota composition and metabolite production. Among dietary components, fiber intake plays a central role by promoting the expansion of SCFA-producing bacteria (Gondalia et al., 2022; Fu et al., 2022). Dietary fiber is fermented by the gut microbiota to produce SCFAs, particularly butyrate, which have been implicated in the regulation of glucose metabolism, insulin sensitivity and inflammatory pathways (Das et al., 2021; Fu et al., 2022).

Mechanistically, metabolic benefits are mediated through the microbiota–SCFA–epigenetic axis. Western-style diets rich in fat and refined sugars suppress microbiota-dependent chromatin remodeling, reduce SCFA availability and impair histone acetylation. In contrast, fiber-enriched diets enhance microbial fermentation, increase SCFA production and promote beneficial epigenetic remodeling within metabolically active tissues (Krautkramer et al., 2016). Accordingly, dietary strategies aimed at stimulating endogenous SCFA production provide a biologically plausible approach for supportive management of T2D. However, clinical studies in humans have shown variable responses, and the magnitude and durability of their effects remain to be fully established.

### 5.2 Engineered probiotics and synthetic biology

Synthetic biology enables development of probiotics engineered to produce metabolites with defined epigenetic activities. These next-generation microbial therapeutics can be programmed to generate bioactive compounds (e.g., butyrate, propionate) to modulate histone deacetylation or DNA methylation. By influencing host transcriptional programs, engineered strains influence metabolic regulation in a targeted manner.

Conventional probiotics, historically applied to manage gastrointestinal disorders (Maftei et al., 2024), have recently been investigated for roles in immune, metabolic and neurobehavioral health (Yoha et al., 2022). However, traditional probiotic formulations face important limitations, including instability in harsh gastrointestinal environments, limited colonization efficiency, lack of tissue specificity and insufficient control over metabolite delivery (Reid et al., 2019).

Emerging technologies, including CRISPR-Cas genome editing, synthetic gene circuits and advanced encapsulation systems, address these constraints by improving microbial stability, functional precision and targeted delivery (Bober et al., 2018). These tools enable controlled therapeutic output and may improve microbial stability and functional precision, positioning engineered probiotics as promising investigational platforms. However, evidence supporting their application in metabolic disease remains largely preclinical, and further studies are required before clinical translation. Although still experimental in metabolic disease, these strategies represent promising avenues with translational potential once several hurdles have been overcome. These include requirements for regulatory approvals, unknown long-term safety, and challenges associated with manufacturing consistency and reliable colonization within the host microbiome.

### 5.3 Postbiotics and metabolite-based therapeutics

An additional and rapidly expanding strategy involves postbiotics, the bioactive metabolites or molecular products derived from microbial activity that exert direct effects on the host. Unlike live probiotic formulations, postbiotic approaches deliver specific compounds such as SCFAs or tryptophan metabolites without requiring bacterial viability.

These metabolites can influence chromatin accessibility, restore disrupted epigenetic marks, and modulate transcriptional programs relevant to metabolic homeostasis (Bhat and Kapila, 2017). Compared to live microbial therapies, postbiotics offer advantages in formulation stability, dosage precision and safety (Zdybel et al., 2025). Nevertheless, evidence supporting the therapeutic use of specific metabolites such as SCFAs, IPA or spermidine in patients with T2D remains limited, and most mechanistic insights derive from preclinical studies. For these reasons, they are being explored as complementary interventions.

The expansion of postbiotic research was facilitated by high-throughput genomic and metagenomic tools, that greatly advanced our understanding of microbial composition and function (Huttenhower et al., 2012). Large-scale microbiome studies established the gut ecosystem as a central regulator of host physiology and a compelling therapeutic target (Lynch and Pedersen, 2016). While early microbiota-based interventions focused primarily on probiotics and prebiotics to modify ecosystem structure, postbiotics represent a mechanistically focused approach that utilizes molecules secreted, transformed or degraded by microbes to act directly on host signaling pathways (Thaiss and Elinav, 2017; Levy et al., 2017).

Collectively, these strategies target upstream regulators of metabolic dysfunction and offer attractive mechanistic opportunities. However, despite encouraging preclinical findings, evidence from human studies remains limited and no advanced-phase clinical trials have specifically evaluated metabolite-mediated epigenetic interventions in T2D. Therefore, these approaches should currently be regarded as complementary to established therapies rather than replacements for conventional glucose-lowering treatments. Rather than focusing on downstream glycemic control, microbiota-centered approaches aim to modify epigenetic and transcriptional networks that influence risk. Dietary fiber interventions provide an accessible foundation, whereas engineered biotics represent emerging precision modalities to expand the therapeutic landscape.

## 6 Conceptual and translational challenges

Despite rapid progress, significant conceptual and translational barriers remain before these insights can be translated to routine application.

### 6.1 Establishing causality beyond association

Many studies associate altered microbial metabolites to changes in host epigenetic landscapes and metabolism, yet associations do not establish causality mechanisms. One example of a causal interaction is microbiota-derived inositol phosphate (InsP₃), generated through bacterial phytate metabolism, which stimulates HDAC3 activity and promotes intestinal epithelial repair and development (Wu et al., 2020). This study provides rare mechanistic evidence linking a microbial metabolite to host chromatin regulation.

Equivalent causal evidence remains limited in metabolic tissues. Furthermore, because many chromatin-modifying enzymes are responsive to diverse metabolic inputs, disentangling the specific contribution of microbiota-derived metabolites from other environmental and host-derived factors remains a major challenge. Emerging programmable epigenetic editing tools offer an opportunity to rigorously dissect these mechanisms. CRISPR/dCas9 platforms have been adapted to induce locus-specific histone modifications, including targeted histone citrullination (Zhang et al., 2024). CRISPRoff systems can establish stable DNA methylation and repressive histone marks without introducing double-strand breaks (Xu et al., 2025). In parallel, engineered lysine acyltransferases allow controlled acetylation and acylation at defined chromatin sites, enabling precise and non-cytotoxic modulation of lysine acylation dynamics (Neumann-Staubitz et al., 2021).

Importantly, these tools modify chromatin states independently of microbial metabolites. They do not require the presence of SCFAs or other bacterial products to induce epigenetic changes. Instead, they directly target specific genomic loci (Fu et al., 2022). Their value in the microbiome–epigenetic field does not lie in mimicking microbial action. Rather, these tools enable testing of whether loci suspected to be metabolite-responsive are functionally relevant. In this way, locus-specific editing can help determine whether microbial metabolites are necessary, sufficient, or merely correlated with diabetes-associated epigenetic alterations.

Several complementary approaches can help strengthen causal inference in microbiome–epigenetic research. For instance, microbiome transplantation studies provide experimental evidence by testing whether transferred microbial communities can alter metabolic outcomes (Wu et al., 2022). Stable isotope tracing has demonstrated direct links between microbiota-derived metabolites and host histone acetylation, while multi-omics approaches can integrate microbial, metabolic, and epigenetic data to reveal mechanistic pathways underlying T2D (Lund et al., 2022; Wang et al., 2021). Together, these approaches help move the field beyond association toward causality.

A related challenge concerns epigenetic mark stability. Some modifications, particularly DNA methylation, can persist through cell division and contribute to long-term transcriptional memory, whereas certain histone modifications are more dynamic and responsive to environmental inputs (Lubin et al., 2011). Determining whether metabolite-induced chromatin changes in diabetes are transient, reversible adaptations or stable ones, remains essential for translation (Sahu and Lu, 2025). Durable epigenetic editing tools provide a means to evaluate the long-term functional consequences of locus-specific modification in metabolic disease models (Wu et al., 2023).

### 6.2 Interindividual variability and epigenetic responsiveness

A second major challenge is substantial interindividual variability in microbiome composition, metabolite production and host epigenetic responsiveness. Pharmacometabolomic profiling in individuals with T2D has demonstrated that metformin responders exhibit distinct metabolite signatures enriched in sphingomyelins, acylcholines and glutathione-related compounds, whereas poor responders display elevated glucose-related metabolites and divergent microbial profiles (Naja et al., 2023). Such heterogeneity complicates development of broadly applicable microbiome-targeted interventions.

Even under controlled conditions, human metabolomic profiles exhibit both stability and fluctuation. Short-term studies indicate that many circulating metabolites remain relatively stable over weeks, supporting their utility as biomarkers (Agueusop et al., 2020). In contrast, longitudinal analyses reveal age-associated shifts, particularly in pathways related to antioxidant defense and urea metabolism (Chaleckis et al., 2016). These findings highlight that metabolic and epigenetic states are influenced by demographic, dietary, environmental and genetic factors.

An additional consideration is the influence of biological sex on microbiota–epigenetic interactions in T2D. Sex hormones can shape gut microbiota composition and function, resulting in differences in microbial communities and their responses to environmental factors (Org et al., 2016; Jasarevic et al., 2016). These observations suggest that biological sex may be an important modifier of microbiota–epigenetic interactions in T2D.

Taken together, variability in microbiota composition, metabolite exposure and chromatin responsiveness suggests that microbiome–epigenome interactions in diabetes are context-dependent rather than uniform. Emerging technologies such as single-cell epigenomics and spatial transcriptomics may help resolve cell-type-specific and spatially localized host responses to microbiota-derived metabolites, providing a more comprehensive understanding of how microbial signals influence epigenetic regulation in complex tissues. Together, these approaches may facilitate the development of more precise and personalized microbiome-based interventions for T2D.

## 7 Conclusion and future directions

T2D is a systemic metabolic disease whose risk and progression are influenced, in part, by the gut microbiota and its metabolite output. Microbial products are increasingly recognized as modulators of host epigenetic programs that govern inflammation, insulin sensitivity and metabolic flexibility. However, these pathways are influenced by multiple interacting signals, including diet, hormones, pharmacological interventions and oxidative stress, highlighting the microbiota as an important contributor rather than the sole driver of metabolic epigenetic regulation.

Across this review, we highlighted how canonical metabolites such as SCFAs, together with emerging classes including tryptophan-derived indoles (e.g., IPA), polyamines and AAA derivatives, converge on chromatin remodeling. Importantly, these effects are not uniform, as metabolite exposure follows a defined anatomical route and is reflected in tissue- and cell-specific epigenetic responses across the colon, liver, pancreatic *β*-cells and adipose tissue. In addition, accumulating evidence suggests that early-life exposures and the maternal microbiome may influence epigenetic trajectories relevant to later metabolic risk.

While these insights provide a strong mechanistic rationale for microbiota-informed approaches, including dietary interventions, postbiotic metabolite delivery and engineered microbial therapeutics, clinical evidence in T2D remains at an early stage. Consequently, these strategies should currently be regarded as investigational adjuncts to established standards of care rather than substitutes for conventional glucose-lowering therapies. Key gaps remain, particularly in distinguishing association from causation, defining the stability and persistence of disease-relevant epigenetic marks and accounting for interindividual variability in microbiome composition and metabolite responsiveness. Priority areas for future research include identifying causal metabolite–epigenetic interactions, characterizing tissue-specific responses and determining the reversibility of diabetes-associated epigenetic changes. Addressing these questions will require longitudinal multi-omics studies, functional validation approaches and experimental tools such as programmable epigenetic editing to establish mechanistic links. In parallel, larger prospective cohorts and well-controlled clinical trials are needed to evaluate the safety, efficacy and translational potential of microbiota-informed interventions. Collectively, integrating microbiome science with epigenetic biology provides a coherent framework for understanding heterogeneity in T2D risk and progression and may ultimately contribute to the development of more precise, mechanism-based strategies for the prevention and management of T2D.
